# A Novel Oral Astaxanthin Nanoemulsion from *Haematococcus pluvialis* Induces Apoptosis in Lung Metastatic Melanoma

**DOI:** 10.1155/2020/2647670

**Published:** 2020-08-26

**Authors:** Hsing-Yu Haung, Yi-Chen Wang, Ying-Chen Cheng, Wenyi Kang, Shang-Hsiu Hu, Dengyong Liu, Chaogeng Xiao, Hui-Min David Wang

**Affiliations:** ^1^Graduate Institute of Biomedical Engineering, National Chung Hsing University, Taichung City 402, Taiwan; ^2^Division of Cardiology, Department of Internal Medicine, Kaohsiung Armed Forces General Hospital, Kaohsiung City 802, Taiwan; ^3^National R&D Center for Edible Fungus Processing Technology, Henan University, Kaifeng 475004, China; ^4^Department of Biomedical Engineering and Environmental Sciences, National Tsing Hua University, Hsinchu 30013, Taiwan; ^5^College of Food Science and Technology, Bohai University, National & Local Joint Engineering Research Center of Storage, Processing and Safety Control Technology for Fresh Agricultural and Aquatic Products, Jinzhou 121013, China; ^6^Food Science Institute, Zhejiang Academy of Agricultural Sciences, Hangzhou 310021, China; ^7^Graduate Institute of Medicine, College of Medicine, Kaohsiung Medical University, Kaohsiung City 807, Taiwan; ^8^Department of Medical Laboratory Science and Biotechnology, China Medical University, Taichung City 404, Taiwan; ^9^College of Food and Biological Engineering, Jimei University, Xiamen 361021, China

## Abstract

Astaxanthin (AST) is a naturally occurring xanthophyll carotenoid having the potential to be used as an anticancer agent; however, the human body has a low bioavailability of AST due to its poor solubility in the water phase. Therefore, we applied D-*α*-tocopheryl polyethylene glycol succinate (TPGS) as an emulsifier and natural edible peanut oil to form a steady oil-in-water (O/W) nanoemulsion loaded with AST (denoted as TAP-nanoemulsion). TAP-nanoemulsions were stable without the droplet coalescence against thermal treatments (30-90°C), pH value changes (over a range of 2.0-8.0), and ionic strength adjustments (at NaCl concentrations of 100-500 mM) measured by dynamic light scattering (DLS). AST within TAP-nanoemulsion was released up to 80% in a simulated intestinal enzymatic fluid *in vitro*, and the overall recovery rate was fairly consistent in the Caco-2 cellular model. In order to further evaluate *in vivo* melanoma inhibitory experiments, we injected the fluorescent-stained B16F10 cells into female C57BL/6 mouse tail veins and treated TAP-nanoemulsion in an oral gavage. qRT-PCR and Western blot demonstrated that TAP-nanoemulsion triggered effectively the apoptosis pathway, including enhancements of cleaved caspase-3 and caspase-9, ataxia-telangiectasia mutated kinase (ATM), and p21WAF1/CIP1 (p21) and decreases of B-cell lymphoma 2 (Bcl-2); cyclins D, D1, and E; mitogen-activated protein kinase (MEK); extracellular signal-regulated kinases (ERK); nuclear factor *κ*-light-chain-enhancer of activated B cells (NF-*κ*B); and matrix metallopeptidase-1 and metallopeptidase-9 (MMP-1 and MMP-9) in both gene and protein expressions. In conclusion, this study suggests that TAP-nanoemulsion with the oral treatment has a positive chemotherapy effect in melanoma with lung metastases *in vivo*. As far as we know, this is the first time to demonstrate that an antioxidant in nanoparticle administration cures lung metastatic melanoma.

## 1. Introduction

*Haematococcus pluvialis* is very famous for its excellent powerful antioxidative property and is one of the widespread freshwater Chlorophyta species from Haematococcaceae family members [1]. *H. pluvialis* is composed of high proteins, low fat, necessary vitamins, and important minerals, including magnesium, iron, calcium, and zinc. Astaxanthin (AST) is a kind of xanthophyll carotenoid, which commonly occurs in natural seafood creatures, such as salmon, trout, shrimp, lobster, and crab; microorganisms; and plants [2]. *H. pluvialis* is frequently observed in temperate climate regions around the world [3]. The high quantity of AST is produced in *H. pluvialis* resting cells, which are secreted and speedily accumulated while the ecological situations develop unfavorably for cell proliferation. This species yielded AST in low accessibility of nutrients, high salinity, and strong bright sunshine irradiation. AST has been reported with many functions like oxidative stress decrease, immune response enhancement, cardiovascular disease treatment, antibacterial property, macular degeneration improvement, anti-inflammatory activity, and certain cancer inhibition [4–6]. However, AST might be limited in aquaculture, medicine, cosmetics, and functional food ingredient business applications due to its low stability in storage and poor solubility in the water phase for the human body. An attractive solving approach is to encapsulate AST within the emulsion delivery system. The nanoemulsion diameter is less than 200 nm and made up of two immiscible liquid phases blended by surfactants and mechanical shear forces. In general, the benefit of nanoemulsion is not only having a higher surface area than the conventional emulsion but also having better bioavailability and physicochemical stability due to the smaller droplet size [7].

Surfactants reduce the interfacial tension of water and oil in the emulsion and combine the hydrophilic group to the aqueous phase and the hydrophobic domain toward the oil phase. The emulsifier forms a stable interface and prevents the oil/water droplet from aggregation [8], and in this study, we apply D-*α*-tocopheryl polyethylene glycol succinate (TPGS) as the emulsifier. TPGS is comprised of the esterification of vitamin E succinate with polyethylene glycol, having advantages of extending the drug half-life in plasma and enhancing cellular uptake capacity. TPGS inhibits ATP-dependent p-glycoprotein transporters, and this unique ability improves the poorly soluble anticancer drug bioavailability, such as paclitaxel. Nowadays, it is widely employed in the hydrophobic drug delivery systems and is a suitable emulsifier to prepare the nanoemulsion as it enlarges the drug encapsulation efficiency and bioavailability. The medium-chain triacylglycerols and hydrolyzed vegetable oil were suggested widely for use in nanoemulsions. We applied natural edible oil and noncytotoxic and stable peanut oil, to raise the embedding concentration of AST.

The gastrointestinal tract secretes lipases to degrade nanoemulsions into diglycerides, monoglycerides, and free fatty acids [9]. The small intestine absorbs lipid components, and the fat-soluble materials need to be broken down by the engulfing action. Small size droplets with high surface areas of nanoemulsions accelerate the degradation ratio and constitute the release rate. In other words, the active agent easily diffuses into the surrounding aqueous environment from an oil phase. After digestion, the chylomicron stimulates bile secretion and gastrointestinal motility. Bile decomposes nanoemulsions as endogenous surfactants and colloidal structures called mixed micelles, and bile with these micelles further dissolve and absorb the free components.

Melanoma is an invasive tumor to metastasize other organs, and its 5-year survival rate is less than 15% [10]. In general, microenvironments and blood flow patterns influence the primary tumor cell spread to target organs. The tight junction with the basement membrane and endothelial cells resists transendothelial cancer migration. Nevertheless, melanoma utilizes the leakage of the vessel wall to spread out [11]. At the present time, nanoemulsion products as anticancer drug vehicles develop the inhibitory efficacies, and we prepare TAP-nanoemulsion to suppress melanoma proliferation and facilitate the apoptosis reactions. This work's purpose is to embed a high concentration and poorly soluble AST in O/W nanoemulsion which is stable at various pH, temperature, and ionic strength surroundings. We test TAP-nanoemulsion cytotoxicity in foreskin fibroblasts and bioavailability in Caco-2 cells and suppression in metastatic melanoma of the lung *in vivo*.

## 2. Materials and Methods

### 2.1. Reagents and Samples

*H. pluvialis* AST extract was purchased from Tianbaoherb Biotech Co., Ltd. (Shanxi Province, China) and Trade Wind Biotech Co. Ltd. (Taiwan). Briefly, the environmentally friendly ethanol supercritical carbon dioxide fluid extraction method was applied to obtain AST extract. TPGS was bought from Wei Ming Pharmaceutical MFG. Co., Ltd. (Taiwan). Peanut oil was obtained from God Bene Enterprise Co., Ltd. (Taiwan). AST (purity > 97%) was used to prepare the standard curve; aprotinin, bile extract porcine, bromo-3-chloropropane (BCP), dimethyl sulfoxide (DMSO), ethylenediaminetetraacetic acid (EDTA), glycerol, hydrochloric acid, *β*-glycerophosphate, pancreatin from porcine pancreas, leupeptin, phenylmethylsulfonyl fluoride, protein assay kit, radioimmunoprecipitation assay buffer (RIPA), sodium chloride, sodium fluoride, sodium hydroxide, sodium orthovanadate, sodium pyrophosphate, Tris-HCl, 3-(4,5-dimethylthiazolyl-2)-2,5-diphenyltetrazolium bromide (MTT), and TRIzol were purchased commercially from Sigma-Aldrich Chemical Inc. (St Louis, MO, USA). Dulbecco's Modified Eagle's Medium (DMEM), fetal bovine serum (FBS), and penicillin-streptomycin-amphotericin (PSA) were obtained from Life Technologies Co., Ltd. (Gibco, Grand Island, NY, USA). All other chemicals within this work were analytical grade. Milli-Q (Merck Co. NJ. USA) water was prepared for all the solutions and nanoemulsions.

### 2.2. Preparation of TAP-Nanoemulsion

AST was dissolved in peanut oil to stir at room temperature by a magnet stirrer for a proper time period, and undissolvable AST was removed via a 0.45 *μ*m membrane filter. Before the preparation of the nanoemulsion, AST concentration in the oil phase was analyzed at the absorbance of 450 nm by a spectrophotometer. TPGS at 0.5% was dissolved in the oil phase at 45°C and uniformly mixed to a soluble status, and then 2.0 mL deionized water was added and shaken to nanosized scale in an ultrasonicator. In the stability assay, we produced different concentrations of TPGS (0.25–1.5%, w/w) of TAP-nanoemulsion using the same method.

### 2.3. Droplet Size Analysis

The droplet size and size distribution of TAP-nanoemulsion was measured by dynamic light scattering (DLS, Nano ZS90, Malvern Instruments Ltd., Worcestershire, UK). This equipment can analyze nanoemulsion droplet size ranging between 0.3 nm and 10 *μ*m. Samples were diluted with a buffer solution (1 : 100) before the analysis to avoid multiple scattering effects. The evaluation of each sample was repeated in triplicate for each experimental condition.

### 2.4. Cryo-Field Emission Scanning Electron Microscopy (Cryo-FESEM)

A drop of dispersion was filled into brass rivets to dive frozen within liquid nitrogen. Samples were stored in liquid nitrogen and transferred into the cryo-FESEM stage (Quorum Technologies, PP3010T, UK) of the microscope (JEOL, JSM-7800F, Japan). The sample was fractured on the cryo-SEM and coated with platinum. Images were acquired at a temperature of −140°C and a voltage of 10 kV.

### 2.5. TAP-Nanoemulsion Stability Assays: Temperature, pH, and Ionic Strength

Physical and chemical stabilities of TAP-nanoemulsions were examined in various experimental surroundings which may be encountered in preparations and storages [12]. Briefly, TAP-nanoemulsion was prepared in 0.5% TPGS, 10% AST, and 20% peanut oil. Fresh TAP-nanoemulsions were put in a water-bath tank at different temperatures (30, 60, and 90°C) for 1 hr, to wait for it to cool to the room temperature before analyzing the resulting samples via DLS. In pH stability assay, TAP-nanoemulsion samples were adjusted to designed pH values (pH 2.0-8.0, phosphate buffer, 5 mM) by adding 0.1 mol/L HCl or 0.1 mol/L NaOH solutions and continuously stirred and then analyzed. Finally, in the ionic strength stability test, we put samples in various salt concentrations (NaCl, 0, 100, 200, 300, 400, and 500 mM). The resulting samples were gently mixed for 30 s, and the samples were put at 5°C for 24 hrs to analyze.

### 2.6. Quantification of AST Amount in TAP-Nanoemulsion System

The concentration of AST in the nanoemulsion system was assayed by UV-visible spectroscopy (Bio-Tek Co., VT, USA) with minor modifications. AST was separated from the nanoemulsion droplet using solvent extraction and quantified from the UV-visible spectrophotometer. 9.8 mL of an organic solvent (methanol : dichloromethane = 1 : 2, v/v) was mixed with 0.2 mL of TAP-nanoemulsion, and the nanoemulsion was completely separated from the oil phase into an aqueous phase. The absorbance was measured at 470 nm and the dichloromethane/methanol solution as a blank control. A calibration curve (R^2^ = 0.9983, data not shown) was calculated for the AST concentration generated from the above solution while the concentration was expressed in mg/mL. Calculating AST encapsulation efficiency in nanoemulsion follows

(1) $$\begin{array}{c} \text{Encapsulation efficiency }\left(\%\right)=\frac{c_{t}}{c_{0}}\times 100, \end{array}$$

where *C*_0_ is the initial concentration of AST in the nanoemulsion, while C_t_ is the concentration of AST at a specific time interval point.

### 2.7. Cell Culture

Human foreskin fibroblasts were gifts acquired from Kaohsiung Medical University (Dr. Su-Shin Lee), and all procedures were approved by the Institutional Review Board (IRB) (Figure S2; KMUHIRB-2014-07-07 (I), Kaohsiung City, Taiwan), and the cells were added with 143 U/mL benzylpenicillin potassium, 10 mM 4-(2-hydroxyethyl)-1-piperazineethanesulfonic acid (HEPES), 1% antiseptic, 10% fetal bovine serum (FBS), 100 *μ*g/mL streptomycin sulfate, and DMEM medium (13.4 mg/mL) [13]. Human colon adenocarcinoma Caco-2 cells were kindly provided by Professor Ping-Shan Lai (National Chung Hsing University, Taichung City, Taiwan). Mouse skin melanoma B16F10 cells were acquired from Bioresource Collection and Research Center (BCRC#60031, Hsinchu, Taiwan). Caco-2 and B16F10 cells were incubated in medium including DMEM with amphotercine 25 *μ*g/mL, 10% FBS, penicillin 100 U/mL, 2 mmol/L l-Glu, and streptomycin 30 *μ*g/mL. Both cells are cultured at 37°C in a 5% CO_2_ humidified atmosphere.

### 2.8. In Vitro Cytotoxicity Study for TAP-Nanoemulsion

Before the experiment, cells are washed with PBS and incubated with DMEM supplement [13, 14]. The cytotoxicity of TAP-nanoemulsion was determined by MTT assay. MTT is a yellow tetrazolium that is absorbed by living cells and changed to a purple color because mitochondrial dehydrogenase is in a less reducing state. Cells were cultured with a density of 8 × 10^3^ cells per well in a 96-well plate. After one-day incubation, fresh DMEM and the nanoemulsion were added to the plate and recultured for 24 hrs. We changed the broth to a medium including 100 *μ*L of MTT (0.5 mg/mL) and incubated at 37°C, 5% CO_2_, for 2 hrs. After that, we discard the medium and added 100 *μ*L of DMSO to dissolve MTT formazan and then quantified the absorbance values (*A*) of the supernatants measured at 595 nm. To determine the cell viability following

(2) $$\begin{array}{c} \text{Cell viability }\left(100\%\right)=\frac{A_{\text{sample}}-A_{\text{blank}}}{A_{\text{control}}-A_{\text{blank}}}\times 100\%, \end{array}$$

where *A*_sample_ is the OD of the sample, *A*_blank_ is the OD of the MTT solution but without cells, and *A*_control_ is the OD of the untreated group.

### 2.9. Measurement of Intracellular Reactive Oxygen Species (ROS) Level in Foreskin

ROS-sensitive fluorescent dye, 2′,7′-dichlorofluorescin diacetate (DCFDA) was used to determine PMA upregulated intracellular oxidative stress level in cells [15]. DCFDA is nonfluorescent, but in ROS presence (when reagent is oxidized), it shows green fluorescence. In order to observe sample antioxidative property, cells were pretreated with and without TAP-nanoemulsion (0-26 *μ*g/mL) for 24 hrs and stimulated by PMA (20 *μ*g/mL) for another 24 hrs. Afterward, it was rinsed with warm phosphate-buffered saline (PBS) buffer, and incubated with 20 *μ*M DCFDA containing PBS at 37°C, 5% CO_2_, for 30 min, to replace a fresh cell medium and to wash cells at least 3 times with PBS. Using trypsin/EDTA cuts away the focal adhesion anchored cell to the culture dish. The cellular fluorescence intensity was analyzed with Guava® easyCyte Flow Cytometers (Merck KGaA, Darmstadt, Germany) at 485 nm excitation and 530 nm emission for 2,7-dichlorofluorescein (DCF) detection.

### 2.10. In Vitro Mimic Digestion Model: Simulated Gastric and Intestinal Fluid

The simulated gastric fluid was made of 7.0 mL HCl and 2.0 g NaCl to set the working volume of 1.0 L to adjust the pH value to 1.2 [16]. The simulated intestinal fluid was dissolved in 2.5 mL of pancreatic lipase solution (60 mg in PBS), 3.5 mL of bile extract (187.5 mg in PBS), and 1.5 mL of calcium chloride (110 mg in water) at pH 7.0, 37°C. TAP-nanoemulsion (2.0 mL) was added to the dialysis bag and put into the simulated gastrointestinal fluid maintained at 37°C and stirred by a magnetic stirrer (RT-10, IKA, Germany) at 100 rpm. At the scheduled time, an aliquot (1.0 mL) was taken and immediately replaced with the same volume of prewarmed fresh PBS. The concentration of AST was analyzed by HPLC to measure three times, and the results are shown as a percentage of AST-released amount over time.

### 2.11. Cellular Uptake and Transepithelial Permeation of TAP-Nanoemulsion

Caco-2 cells (1 × 10^5^ cells/2.4 cm^2^ insert) were seeded in a transwell chamber (0.4 *μ*m pore size, 24 mm diameter; Costar, Kennebunk, ME, USA). The cell culture media were changed every two days, and we evaluated the integrity of monolayers by a Millicell ERS-2 epithelial cell volt-ohm meter (Taiwan Instrument Co., Ltd, Taiwan) to determine the transepithelial electrical resistance (TEER) by [17]

(3) $$\begin{array}{c} \text{TEER }\left(\Omega \cdot {\text{cm}}^{2}\right)=\left({\text{ohm}}^{1}–{\text{ohm}}^{2}\right)\times A, \end{array}$$

where *A* is the surface area of the insert (4.4 cm^2^), ohm^1^ is the resistance of the insert with cells, and ohm^2^ is the resistance of the insert with cell culture medium only.

After the incubation, the resistance of cells achieved above 500 *Ω*/cm^2^ could be used for performing all the experiments. To analyze the cellular uptake of TAP-nanoemulsion in vitro, the medium was changed to fresh DMEM for 24 hrs before the infiltration experiment. The monolayer cells were washed twice with PBS (pH 7.4, 37°C), and it was cultured with TAP-nanoemulsion overnight at 37°C in 5% CO_2_ atmosphere. TAP-nanoemulsion was gently removed after the incubation, and the resulting solution was loaded into a 6-well transwell plate. To analyze AST permeability, 0.5 mL aliquot was added to each test solution (1.66, 3.22, 6.64, 13.3, and 26.6 *μ*g/mL AST in TAP-nanoemulsion) in the top side of the transwell plate (donor chamber). The receiving chamber obtained 0.5 mL of medium, and all samples were cultured in a 5% CO_2_ incubator overnight. After the culture, the apical and receiving chamber solutions were gathered and analyzed by UV-visible spectroscopy.

### 2.12. Animal Model

In the research, the animals conformed to the guidelines of the American Society of Physiology for animal care and use, approved by the National Chung Hsing University and Institutional Animal Care and Use Committee (Figure S3; IACUC number: 105-141). C57BL/6 female mice (6-7 weeks) were bought from BioLASCO Experimental Animal Center (Taiwan Co., Ltd.). All mice were kept in plexiglas cages in a temperature-controlled room (22 ± 1°C) with a light/dark (12/12 hrs) schedule and free to obtain food and water [18]. Fifteen C57BL/6 mice were randomly divided into 3 groups: vehicle control (n = 3), tumor only (n = 6), and TAP-nanoemulsion treatment (n = 6), after 7-day prebreeding.

### 2.13. Tumor Development and TAP-Nanoemulsion Regimen Administration

B16F10 cells were cultured in DMEM and stained with PKH26, and all experiments were carried out in cells cultured less than 15 passages. We injected the fluorescent stained cells (1 × 10^5^ cells within 200 *μ*L of PBS) into female mouse tail veins twice a week. Two weeks after tumor injection, the mice were treated with the following conditions: vehicle control group (without treatment), tumor-only group (0.2 mL normal saline per day), and TAP-nanoemulsion group: 10 mg TAP-nanoemulsion per every other day by an oral gavage. The body weights of all mice were recorded every twice a week to check that the mice are healthy. Tumor growth inhibition was measured by IVIS spectral imaging system (Caliper Life Sciences, Hopkinton, MA, USA). After the 35^th^ day, all mice were sacrificed by cervical dislocation, and the lung, spleen, heart, kidney, and liver organs were gathered at the end of this study.

### 2.14. Quantitative Real-Time Reverse Transcription Polymerase Chain Reaction (qRT-PCR) Analysis

qRT-PCR consists of an exclusive primer probe to generate the fluorescence signal. It uses a fluorescent detection technique to sense each cycle by a StepOnePlus™ System (Thermo Fisher Scientific Inc., USA). It detects and records the fluorescent intensity per cycle and calculates achieving the real-time quantitative data. We applied TRIzol™ (Invitrogen Co., CA, USA) to extract RNA from the lung tissue, and subsequently, a reverse transcription kit (Takara, Japan) generated DNA. Primers for qRT-PCR are listed in Table S1. First, the sample was heated to form a single strand of DNA. Second, primers were bound to form a double-stranded DNA (dsDNA). Third, SYBR Green dsDNA were combined by SYBR green Plus reagent kit (Roche, Basel, Swiss), releasing the fluorescence. A detection of fluorescent signals during the elongation or annealing phase of each cycle was detected [4, 19]. The expression levels of target genes were normalized with *α*-tubulin levels using the 2^-*ΔΔ*Ct^ method.

### 2.15. Western Blotting of Lung Organization

Lung tissues were homogenized with RIPA buffer and centrifugated of the lysate at 18,000 g for 30 min. The supernatant was analyzed by BCA (bicinchoninic acid) protein assay kit (Sigma-Aldrich Co., St. Louis, MO, USA) [20, 21]. The samples (20 ng) were qualified by Western blotting protein electrophoresis on sodium dodecyl sulfate (SDS). The 8-10% SDS gels were transferred to polyvinylidene fluoride (PVDF) membranes. Next, PVDF membranes were added into Bcl-2, cleaved caspase-3 and caspase-9, ataxia-telangiectasia mutated kinase (ATM), p21WAF1/CIP1 (p21), cyclins D and E, mitogen-activated protein kinase (MEK), extracellular signal-regulated kinases (ERK), nuclear factor kappa-light-chain-enhancer of activated B cells (NF-*κ*B), and matrix metallopeptidase-1 and metallopeptidase-9 (MMP-1and MMP-9, respectively) antibodies at 4°C for 24 hrs. It was added into biotinylated secondary antibodies at 25°C for 1 hr. Finally, it was detected by enhanced chemiluminescence (ECL) detection reagent (PerkinElmer, USA; ECL1 : ECL2 = 1 : 1) exposing it to X-rays for a specific period of time to obtain the vision. Dyeing images can be visualized via a commercially available image system. We took *α*-tubulin as our experimental loading control.

### 2.16. Statistical Analysis

All results within each experiment were in triplicate and presented as a mean ± standard deviation (SD). All data were compared multiple times by Student's t-test for statistical analysis. Significant difference (∗) was defined as p < 0.05.

## 3. Results and Discussion

### 3.1. Characterization of TAP-Nanoemulsion in Different Surroundings

#### 3.1.1. Effects of Various Emulsifier Concentrations on TAP-Nanoemulsion Droplet Diameters

**Figure 1:**
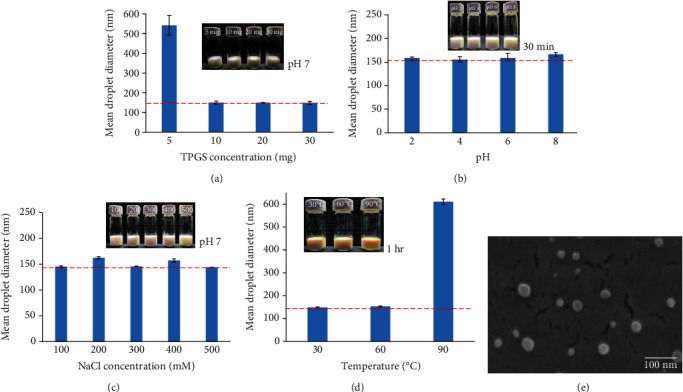
The influences on the physical stabilities of TAP-nanoemulsion. (a) Different TPGS concentrations, 2.5–15.0 mg/mL; (b) various pH values, pH 2.0–8.0; (c) diverse NaCl concentrations, 0–500 mM; and (d) various temperatures, 30–90°C. (e) Cryo-FESEM images of TAP-nanoemulsion.

It is well known that oil and water are immiscible because of polar properties, and the interfacial tension between two phases is fairly high. In order to successfully prepare emulsions, surfactants adsorbed at the boundary reduce the interfacial tension and droplet size [22]. We made nanoemulsions in different emulsifier concentrations and measured the particle size changes in Figure 1(a). TAP-nanoemulsion with 5 mg TPGS had the biggest size distribution and the mean particle radius was 542.1 ± 49.5 nm (polydispersity index, PDI: 0.251). Conversely, increasing TPGS concentration to 10 mg, the average droplet size decreased 3.5 times and the mean particle radius was 150.5 ± 7.32 nm (PDI: 0.239). The average droplet sizes of the samples with treatments at 20 and 30 mg were 155.0 ± 40.8 and 145.6 ± 27.7 nm, respectively. It was insignificantly altered to compare with over 10 mg dosage, and the system was pretty stable at least six months better than 5 mg treatment. Therefore, as the emulsifier concentration increased from 0 to 10 mg TPGS, the globule size reduced to around 150 nm and the system stability was improved.

#### 3.1.2. Effects of Various pH Values on TAP-Nanoemulsion Droplet Diameters

In beverage and food, the pH value of the aqueous phase is widely distributed, from acidic soft drinks to minor alkaline nutritional beverages. We investigated the pH value influence on TAP-nanoemulsion physical stability stored at 25°C for one day in Figure 1(b). The nanoemulsion with 10 mg TPGS was stable in different pH environments, and the droplet size remained constant (~150 nm), and there was no phase segmentation phenomenon and droplet aggregation. At pH 8.0, the droplet size showed only a little increase, indicating TAP-nanoemulsion was stable at pH 6.0. With pH at 2.0 and 4.0, TAP-nanoemulsion droplet size was 154.0 ± 8.6 and 155.3 ± 4.9 nm, respectively. The results indicated TAP-nanoemulsion was stable at a wide pH range from 2.0 to 6.0 because of the steric repulsion.

#### 3.1.3. Effects of Various Ionic Strengths on TAP-Nanoemulsion Droplet Diameters

Analyzing TAP-nanoemulsion stability for ionic strength is important since many emulsified foods and beverages may contain some minerals that affect the final product stability and appearance. TAP-nanoemulsion permanence was investigated at different NaCl concentrations (0, 100, 200, 300, 400, and 500 mM) at pH 7, 25°C in Figure 1(c). The electrostatic repulsion was sufficient to ensure both hydrophobic attractiveness and Van Der Waals forces were low at all designed salt concentrations. We added NaCl to decrease the electrostatic repulsions between nanoparticles which led to the O/W droplet becoming unstable. As NaCl concentration increased to 500 mM, the droplet size did not significantly vary, and its average particle radius was 143.8 ± 0.7 nm (PDI: 0.144). The spatial repulsive force of TAP-nanoemulsion droplets was strong enough to keep droplets steady even at high NaCl solution dosage of our experimental conditions.

#### 3.1.4. Effects of Various Temperatures on TAP-Nanoemulsion Droplet Diameters

We designed this nanoemulsion model to release AST after being attached to the digestive enzymes in the gastrointestinal system *in vivo*. As we know, the mammal creature temperature range is above 30°C (34-38°C), and we also consider the temperature changes at the manufacturing, shipping, and storage processes. Accordingly, we proposed the temperature stability between 30 and 90°C as the constancy assay (Figure 1(d)) to avoid the TAP-nanoemulsion quality lost during the whole process. After it was incubated at 30°C and 60°C for 1 hr, the particle size did not notably vary. The droplet size markedly increased at 90°C, and the inference of unstable nanoemulsion was due to the droplet coalescence and aggregation to weaken the interfacial tension compared to the same in recent studies. We observed the solution color was a kind of orange-red due to AST separation from the oil phase at high temperature. Prior studies also found that carotenoid degrades quickly when stored at high temperatures [22], indicating carotenoids were unstable due to biochemical demotion. TAP-nanoemulsion was destructed at 90°C, showing temperature had a major effect on the short-term thermal stability of the nanoemulsion, and AST release increased when the temperature increased.

#### 3.1.5. Cryo-FESEM Images of TAP-Nanoemulsion

The microstructure of TAP-nanoemulsion was observed by electron microscopy in Figure 1(e). One advantage of using cryo-FESEM was that the liquid dispersion could be frozen and viewed in the solid state. The micrograph of TAP-nanoemulsion revealed spherical droplets in the nanometer scale range which was in agreement with the size data determined by DLS as above.

### 3.2. Cytotoxicity Evaluations on TAP-Nanoemulsion System

**Figure 2:**
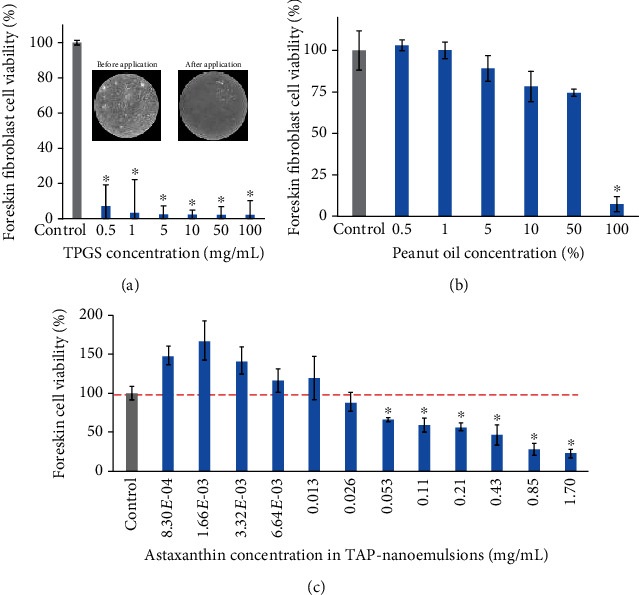
The cellular viability of foreskin fibroblasts was measured by MTT assay after (a) TPGS, (b) peanut oil, and (c) TAP-nanoemulsion treatments for 24 h. The data was represented with mean values ± SD of three independent experiments performed. ^∗^p < 0.05 as compared with the vehicle control group.

Any latent chemoprotective substance should be first evaluated for its sensitivity and safety, and we assessed our raw materials via MTT assay on human primary foreskin fibroblasts [1, 4]. The cellular viability was less than 10% after treatments with TPGS over 1 mg/m in Figure 2(a), and the surfactant was cytotoxic to cells. It was a normal phenomenon that adding a surfactant directly to the culture medium will cause cell membrane lysis and cell death. In previous studies, TPGS was used as an anticancer agent to enhance cellular uptake. Fibroblasts had cell growths greater than 90% at peanut oil concentrations lower than 50% (v/v) in Figure 2(b), and the peanut oil was not toxic. To cultivate cells with all peanut oil (100%, v/v) forces cells to suffocate and die. Finally, cells were incubated with TAP-nanoemulsion at suitable concentrations in Figure 2(c). After AST dosage within the nanoemulsion reached to 53 *μ*g/mL, the cell viability declined gradually. We believed high TPGS concentrations induce cell death due to the above reason. In order to avoid the experimental errors inflicted by undesired toxic effects, the highest dosage of TAP-nanoemulsion was set at 53 *μ*g/mL.

### 3.3. Antioxidative Assay of TAP-Nanoemulsion System

**Figure 3:**
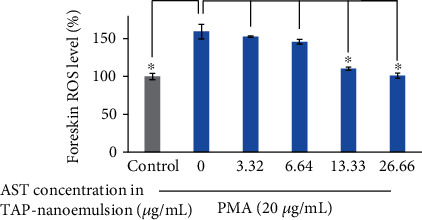
The ROS production percentage was enhanced by PMA (20 *μ*g/mL) and reduced via TAP-nanoemulsion administration measured by flow cytometry.

Antioxidants are important agents for foods and cosmetics because of the core functions in reducing the radical-induced degradation and scavenging free radicals of living cells and tissues. We already published several papers to demonstrate that 3S, 3′S-astaxanthin is the most powerful antioxidant to scavenge ROS all over the world [1, 2, 6]. It has a powerful capacity to scavenge hurt-free radicals than vitamins and other natural pure compounds, especially in human healthy rejuvenation applications. For measuring the antioxidative properties on the testing samples of interest, the cellular experimental platform was carried out in practice. DCFDA staining is a typical quantitative method for the intracellular H_2_O_2_ amounts to survey oxidative stresses [1]. To determine whether TAP-nanoemulsion suppresses oxidations, we investigate ROS generations in cell-based foreskin fibroblasts. PMA is an inducer for endogenous superoxide secretions, and PMA-activated cells produced ROS which emitted the fluorescent intensity to be detected [23]. In Figure 3, it was demonstrated that TAP-nanoemulsion gradually reduced oxidative stresses in a dose-dependent manner. The fluorescence potency of DCFDA was reduced from 159.35% to 101.00% (58.35% reduction) at 0 and 26.66 *μ*g/mL AST-nanoemulsion at 20 *μ*m/mL PMA presence. We discovered that TAP-nanoemulsion inhibited the production of cellular ROS successfully.

### 3.4. TAP-Nanoemulsion Bioaccessibility Measurement In Vitro

**Figure 4:**
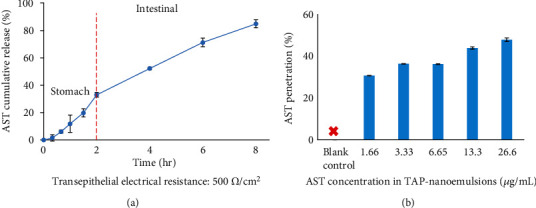
(a) Bioaccessibility of TAP-nanoemulsion after each step of an *in vitro* gastrointestinal mimic digestion. (b) Permeability of TAP-nanoemulsion across the Caco-2 monolayer with various concentrations (0–26.6 *μ*g/mL). Each value was represented as the mean values ± SD (n = 3).

During the digestion process, TAP-nanoemulsion passed through three phases, including the oral, gastric, and small intestine stages. It should be noted that the size and interface characteristics of nanoemulsion might be changed during component assimilations [5, 6]. Our sample was in an aqueous gavage form (hence, the chewing procedure was irrelevant), and the oral residence time was ignored. Moreover, it did not contain starch, meaning the main enzyme did not work on amylase and saliva. The bioavailability of TAP-nanoemulsion was evaluated via incubating with the simulated digestive fluid environmental model, and AST kinetic release was monitored by the cumulative amount for 8 hrs in Figure 4(a). The gastrointestinal release profile exhibited two-stage drug discharge behaviors in the stomach and intestines. Initially, AST was released slightly and followed to speed up in the first phase. As for the gastric fluid digestion in vitro, we observed that the cumulative release sum of AST was less than 35% within 2 hrs. The small intestine is a digestive tract responsible for physiological activity and drug absorption. We found that AST was slowly released from simulated intestinal surroundings, and the released amount was higher over 80% after another 6 hrs in the second digestive phase. A reasonable explanation for this phenomenon was that lipases leisurely penetrated approaching the oil droplets to destroy the TAP-nanoemulsion conformational structure. In addition, bile adsorption took place in the oil/water interface contributing to the lipase attack on the lipid hydrolysis [24].

### 3.5. AST Uptake in Caco-2 Cell from TAP-Nanoemulsion

In our research, TEER calculated values were confirmed whether the Caco-2 confluent Transwell® monolayer culture was suitable for the permeation experiments [25]. The values increased while the culture time was extended, and it reached over 500 *Ω*·cm^2^ after the incubation, and the data is shown in Figure 4(b). AST was retrieved from the basolateral cell and top compartment after the Caco-2 cells were cultured for 24 hrs. The overall recovery rate of different AST-nanoemulsion concentrations raised fairly ranging from 30.7 ± 0.04% (1.6 *μ*g/mL AST) to 47.9 ± 0.86% (26.6 *μ*g/mL AST). AST imbalanced recovery loss might be due to AST metabolic conversion and chemical biodegradation occurrence during transepithelial infiltration. It was reported that the diameter of nanoemulsion less than 200 nm was delivered through the epithelial layer by the passive transport [26]. TAP-nanoemulsion with smaller particle size was able to penetrate the cell membrane efficiently compared to the conventional emulsion. Moreover, the smaller droplet had an extensive surface area to volume ratio resulting in an elevated cellular uptake. AST may have to be released from the capsule matrix by mechanical, chemical, and enzymatic processes that take place in the mouth, stomach, and small intestine. The released AST may be merged into droplets formed by coingested lipids within the mouth and stomach. Dietary lipids, such as triacylglycerols (TAG), undergo digestion within the stomach and small intestine due to the presence of gastric and pancreatic lipases leading to the formation of free fatty acids (FFA) and monoacylglycerols (MAG). These surface-active lipid digestion products interact with endogenous surface-active lipids (bile salts and phospholipids) to form mixed micelles, vesicles, and other colloidal structures [27]. These colloidal structures are capable of solubilizing lipophilic bioactive components (such as *β*-carotene) and transporting them to the epithelium cells where they are absorbed.

### 3.6. TAP-Nanoemulsion Inhibits Lung Metastatic Melanoma Proliferation In Vivo

**Figure 5:**
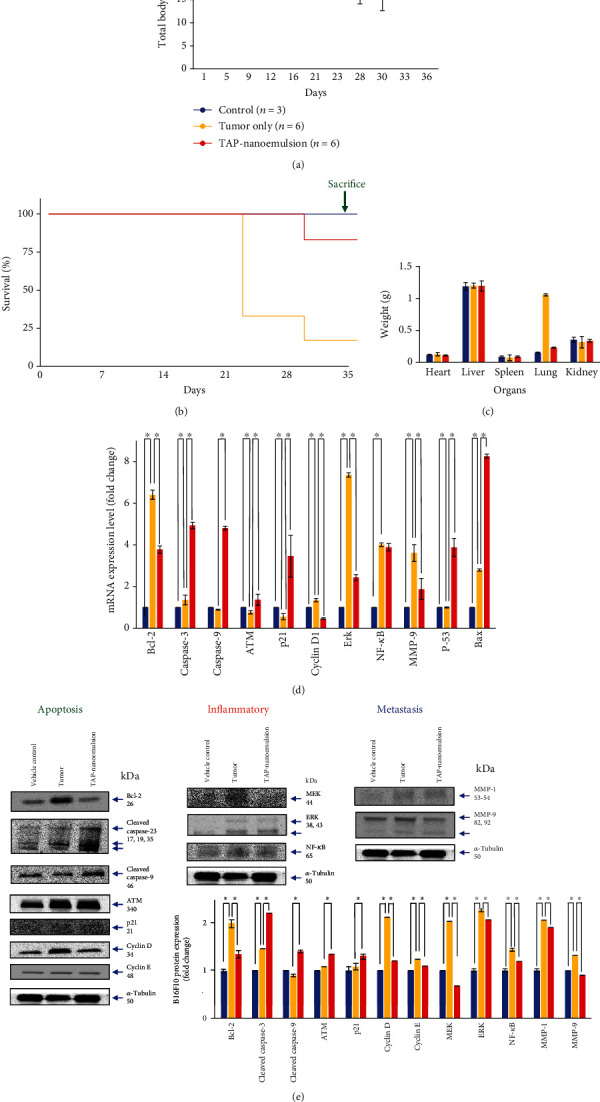
*In vivo* behavior, TAP-nanoemulsion administrated against metastatic melanoma in the lung of B16F10-bearing C57BL/6 mice. (a) Changes in mouse body weight after different treatments. (b) Mouse morbidity-free survival efficacy with different treatments following the vehicle control, tumor-only, and TAP-nanoemulsion groups. (c) The evaluations of organ weights observed from the three groups. (d) The mRNA expressions associated with the three groups in qRT-PCR assay. (e) TAP-nanoemulsion inhibited lung metastatic melanoma through induction of apoptosis-related proteins by Western blotting. We took *α*-tubulin as our experimental loading control. Data are representative of 3 experiments. Blue: blank control; yellow: tumor only; red: TAP-nanoemulsion treatment groups. ^∗^p < 0.05 as compared with the tumor-only group.

The oral administration strategy is preferred over a variety of other administration routes of medicine delivery due to the many benefits it exhibits. The oral dosage forms include liquids, tablets, capsules, and granules. The advantages of the oral liquid form are the most convenient way of drug administration. These advantages include good patient compliance, high versatility, prolonging the time of curative effect, reducing the fluctuation of the blood concentration, pain avoidance, ease of ingestion, improving the compliance of the medication, reducing the number of administrations, decreasing the side effects, and superior safety to accommodate different types of medicines. At first, we designed TAP-nanoemulsion as nutritional supplements. Nanoemulsions have shown the potentials to deliver various kind of drugs (hydrophilic, lipophilic, and amphiphilic agents) through many routes like oral, transdermal, ophthalmic, and parenteral modes. When the agent is directly injected to the site of intravenous (IV) injection, some medicines may cause local irritation. These pharmaceuticals, as well as certain cosolvents in aqueous solutions, induce the phlebitis, an inflammation of a vein leading to pain or redness. Considering the convenience, practicality, and safety, we planned the accumulation by oral administration. We assessed the anticancer potential of TAP-nanoemulsion in a metastatic melanoma C57BL/6 mouse model. Mice were anesthetized with 3.5% isoflurane (Abbott Scandinavia AB, Stockholm, Sweden) and injected with B16F10 cells (1 × 10^5^ cells within 200 *μ*L PBS) through the tail vein at each concentration of 150 mg/g mouse body weight [7]. Anesthesia is kept by 2% isoflurane during bioluminescence scanning using an IVIS spectral imaging system to observe the red signal strength enhancement which corresponded with the tumor development. The result was quantified by the software Living Image 4.3, and images were obtained at 2 min intervals. The region of interest (ROI) is used to delineate the signal and made measurements. The signal intensity (radiation) was analyzed in photons/s/cm^2^/steradian (p/s/cm^2^/sr). The melanoma-only group showed a strong metastasis consequence of B16F10 in the thorax, especially in the lung organ. After one week, all mice presented melanoma invasion results in the melanoma-only group. Until about three weeks, the strong metastatic developments were observed, and all mice were dead in the no-treatment group. It is about two weeks from the beginning to start the oral administration after tumor injection, and the mice were treated with the vehicle control or TAP-nanoemulsion. TAP-nanoemulsion was conducted to the experimental group at 10 mg/kg by the oral route and did not generate any significant changes and problems in normal defecation, posture, expression, respiration, skin shedding, and yellowing of the hair. The mice's body weight was measured every week during the experiment time period. As shown in Figure 5(a), the body weight of the TAP-nanoemulsion group was significantly enhanced over 30 days, and the volume of the mouse tumor was reduced compared to the 0 day between the three groups (Figure S1). Notably, the TAP-nanoemulsion group demonstrated longer survival periods, and only 1/4 of mice died within 35 days in Figure 5(b). The heart, liver, spleen, lung, and kidneys were cut from the sacrificed mice and weighed (Figure 5(c)). The weight of the lung from the tumor-only group compared with that from the vehicle control group was increased because the extra tissue of lung metastatic melanoma. On the other hand, after being treated with TAP-nanoemulsion in an oral form, the metastatic melanoma of the lung was reduced appreciably. In the tumor-only group, the average lung weight was 1.06 ± 0.06 g, whereas the TAP-nanoemulsion group suppressed the lung melanoma proliferation weights to 0.25 ± 0.05 g. There was no major difference in other organ weights among the three groups, suggesting that TAP-nanoemulsion had no significant toxic effects on these parts. Our previous study confirmed that AST restrained the formation of melanoma [15], and in this case, we also inhibited B16F10 cellular melanoma with lung metastases successfully.

Based on our previous findings, we discovered AST suppressed melanoma proliferation via apoptotic mechanism *in vitro* and in a xenograft model [6, 7], and the lung metastatic melanoma inhibition by the oral TAP-nanoemulsion administration within this work was demonstrated. Bcl-2 family proteins are well known as major regulators of the mitochondrial apoptosis mechanism. We found that *Bcl-2* decreased in the TAP-nanoemulsion treatment group in Figure 5(d), and it was assumed that TAP-nanoemulsion increased downstream representative apoptotic expressions of *caspase-3* and *caspase-9* which were proteolytic mRNA playing necessary roles in the programmed cell death. The *ATM* gene provides instructions for making a protein that is located in the nucleus, where it helps to control the cell growth and division rates. In particular, the nanoemulsion promoted the expression of *ATM* in the mouse lungs compared to the other two groups. The activities of cyclin-dependent kinases are positively regulated by cyclins and negatively adjusted by cyclin-dependent kinase inhibitors. The cyclin-dependent kinase inhibitor p21 triggers cell cycle arrest to stop at the G1 phase in many stimulus responses [28]. It was important for the transition stage from G1 to S, and the suppression of *cyclin D1* reduced the hyperplasia of tumor cells. Extracellular signal-regulated protein kinase (ERK) signaling communicates to a neighboring protein by adding phosphate groups to play a switch position in the cancer process and is a required pace in the assistance of melanoma. Nuclear factor *κ*-light-chain-enhancer of activated B cell (NF-*κ*B) not only facilitates tumor initiation/proliferation/development, augments angiogenesis, inhibits apoptosis, and resists drugs but also causes epithelial-mesenchymal transition which assists distant metastasis. Most human cancers have a constitutive of NF-*κ*B because of the numerous oncogenic mutations and inflammatory microenvironments [29]. TAP-nanoemulsion inhibited mRNA expressions of *ERK* with significant difference and minor effect on *NF-κB*. The famous migratory mRNA of matrix metalloproteinase-9 (*MMP-9*) was lower in the TAP-nanoemulsion treatment group than in the tumor-only group. The experimental data showed that it had a positive potential to suppress the invasion and metastasis of melanoma in the lung.

Following the gene expression analysis changes measured by qRT-PCR *in vivo*, we believed that our sample induced lung metastatic melanoma apoptosis due to the invasive suppression. To determine the involvements of signal transduction pathways in TAP-nanoemulsion cancer inhibition, further assays were shown to survey the protein expressions in Figures 5(e) and S4. Western blotting was used to measure the related protein expressions. TAP-nanoemulsion inhibited Bcl-2, cyclin D, ERK, NF-*κ*B, and MMP-9 that exhibited similar resultant trends as qRT-PCR consequences. Dysregulated cyclin E activity causes cell lineage-specific abnormalities, such as impaired maturation, apoptosis, and senescence [30]. MEK is often overactive in some cancers. Once it is blocked, tumor growth is controlled and apoptosis is to take the field. The MMP family is related in the breakdown of all kinds of extracellular matrixes in the normal physiological processes. MMPs are recognized to be implicated with the tumor adhesion and dispersion, the cytokine and chemokine inactivation, the release of apoptotic ligands, the angiogenesis, and the cleavage of cell surface receptors [7]. In particular, the expressions of three proteins (cyclin E, MEK, and MMP-1) were reduced with the addition of TAP-nanoemulsion. Overall, we confirm that TAP-nanoemulsion effectively reduced the growth of melanoma and induced lung metastatic melanoma apoptosis in mRNA and proteins.

## 4. Conclusions

**Figure 6:**
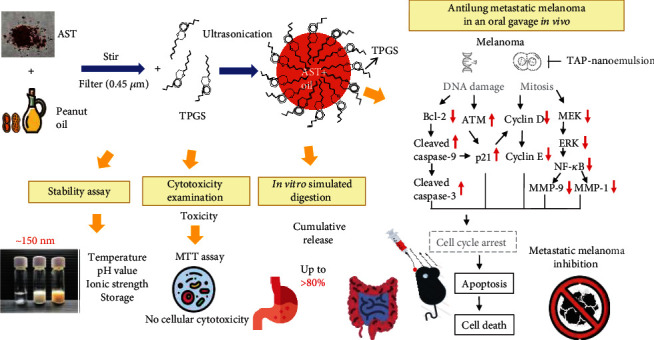
Proposed schematic diagram of TAP-nanoemulsion biofunction.

Based on the above results, we successfully manufactured a nanoemulsion loaded with AST and peanut oil, and TPGS as the emulsifier effectively stabilized the O/W nanoemulsion against various environment stresses. TAP-nanoemulsion had a good antioxidative stress in a dose-dependent manner to reduce the production of cellular ROS. In the physiologic mimic surrounding, TAP-nanoemulsion demonstrated a high-quality oral release characteristic and reasonable stability *in vitro*. TAP-nanoemulsion triggered apoptosis of human malignant melanoma in the lung by inhibiting Bcl-2, cyclins D1 and E, NF-*κ*B, ERK, MEK, and MMP-1 and MMP-9 and increasing cleaved caspase-9 and caspase-3, ATM, and p21. We are the first group to present that TAP-nanoemulsion had the potential clinical application for antioxidation and metastatic melanoma suppression *in vivo* in Figure 6.

## Data Availability

The data used to support the findings of this study are available from the corresponding author upon request.

## Supplementary Materials

Supplementary Materials Table S1: the primer sequences used in this study. Figure S1: tumor developments and TAP-nanoemulsion regimen administration in vivo. Figure S2: IRB certificate of human foreskin fibroblasts [KMUHIRB-2014-07-07 (I)]. Figure S3: IACUC certificate of animal examination (IACUC number: 105-141). Figure S4: TAP-nanoemulsion inhibited lung metastatic melanoma through induction of apoptosis-related proteins by Western blotting verification.
