# Supplementary material for: A Novel Oral Astaxanthin Nanoemulsion from Haematococcus pluvialis Induces Apoptosis in Lung Metastatic Melanoma
Source: Oxid Med Cell Longev. 2020 Aug 26;2020:2647670. doi: 10.1155/2020/2647670 (PMC7471791; doi:10.1155/2020/2647670)
Supplement: Supplementary Materials — Table S1: the primer sequences used in this study. Figure S1: tumor developments and TAP-nanoemulsion regimen administration in vivo. Figure S2: IRB certificate of human foreskin fibroblasts [KMUHIRB-2014-07-07 (I)]. Figure S3: IACUC certificate of animal examination (IACUC number: 105-141). Figure S4: TAP-nanoemulsion inhibited lung metastatic melanoma through induction of apoptosis-related proteins by Western blotting verification. [file 2647670.f1.pdf]

Research Article

A novel oral astaxanthin nanoemulsion induces apoptosis in lung metastatic melanoma

Hsing-Yu Haung [1,†], Yi-Chen Wang [2,†], Ying-Chen Cheng [1], Wenyi Kang [3], Dengyong Liu [4], Chaogeng Xiao [5], Hui-Min David Wang [1,6,7,8*]

1 Graduate Institute of Biomedical Engineering, National Chung Hsing University, Taichung City 402, Taiwan

2 Division of Cardiology, Department of Internal Medicine, Kaohsiung Armed Forces General Hospital, Taichung City 411, Taiwan.

3 National R & D Center for Edible Fungus Processing Technology, Henan University, Kaifeng 475004, China

4 College of Food Science and Technology, Bohai University; National & Local Joint Engineering Research Center of Storage, Processing and Safety Control Technology for Fresh Agricultural and Aquatic Products, Jinzhou 121013, China

5 Food Science Institute, Zhejiang Academy of Agricultural Sciences, Hangzhou 310021, PR China

6 Graduate Institute of Medicine, College of Medicine, Kaohsiung Medical University, Kaohsiung City 807, Taiwan

7 Department of Medical Laboratory Science and Biotechnology, China Medical University, Taichung City 404, Taiwan

8 College of Food and Biological Engineering, Jimei University, Xiamen, 361021, China

† These authors contributed equally to this work.

* Correspondence: Hui-Min David Wang, Ph.D. davidw@dagon.nchu.edu.tw; Mobil: 886-935753718; Tel.: 886-4-22840733#651; Fax: 886-4-22852242

Table S1. The primer sequences used in this study.

Bcl-2
Forward: 5′-GTATTGGTGAGTCGGATTGCAA-3′
Reverse: 5′-TGCTGCATTGTTCCCGTAGAG-3′
Caspase-9
Forward: 5′-TCTGGGACGCTCTGCTGAGT-3′
Reverse: 5′-CTCGGGTCTCAAGGTCTGTGA-3′
Caspase-3
Forward: 5′ -CAGTGGGACTGATGAGGAGATG-3′
Reverse: 5′ -CGACCCGTCCTTTGAATTTC-3′
ATM
Forward: 5′-GACAACAAGAAGGAGTCGAATAACC-3′
Reverse: 5′-GCTTACTTCTGATGGAGTGGATGAT-3′
p21
Forward: 5′-TCTGAGCGGCCTGAAGATTC-3′
Reverse: 5′-CAATCTGCGCTTGGAGTGATAG-3′
Cyclin D1
Forward: 5′-GAGGCGGATGAGAACAAGCA-3′
Reverse: 5′-CTCCCAGCAGCTACCATGGA-3′
NF-κB
Forward: 5′-TATGTGTGTGAAGGCCCATCA-3′
Reverse: 5′-ACCACTGAACGATAACCTTTGC-3′
ERK
Forward: 5′-CGACCACGTTCAGTTTCTCATC-3′
Reverse: 5′-TCTTCGTTCACAGCTAGGTTGCT-3′
MMP-9
Forward: 5′-TCGCGTGGATAAGGAGTTCTC-3′
Reverse: 5′-GGAAACTCACACGCCAGAAGA-3′

Figure S1. Tumor developments and TAP-nanoemulsion regimen administration *in vivo.*

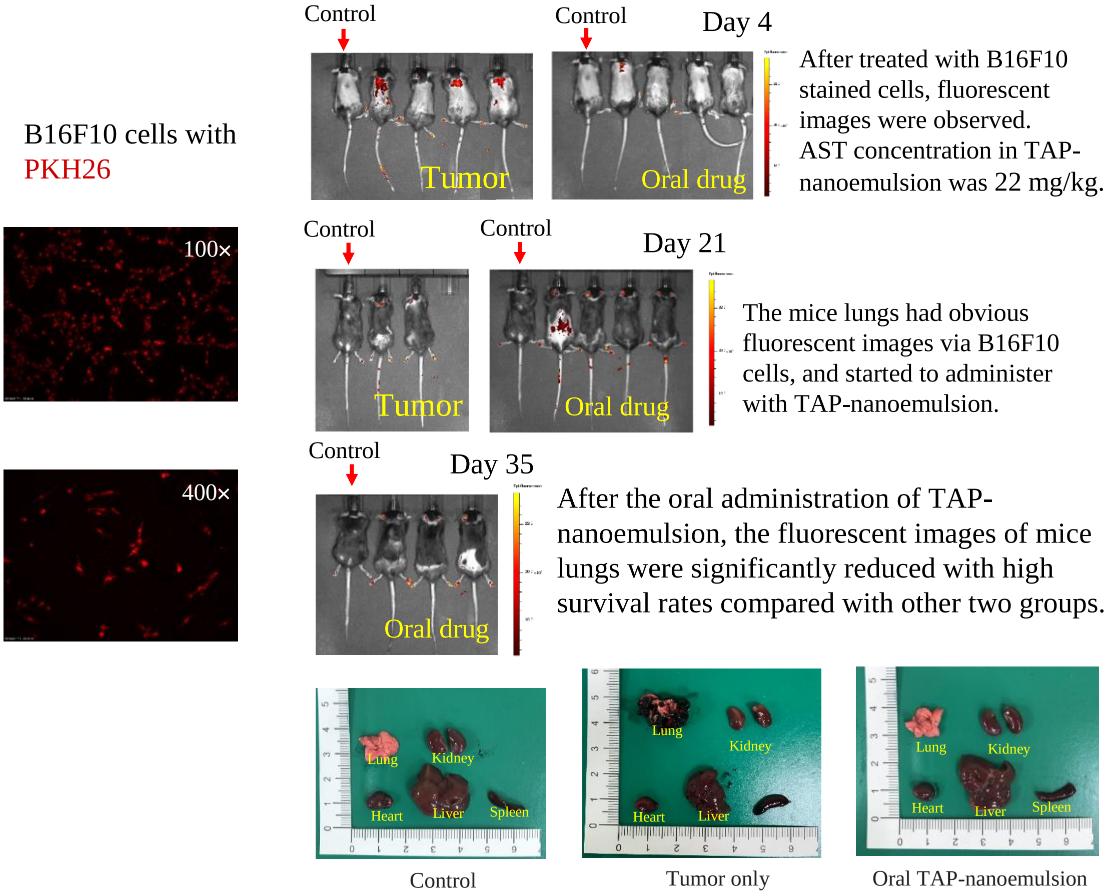

Figure S2. IRB certificate of human foreskin fibroblasts [KMUHIRB-2014-07-07 (I)].

高雄醫學大學附設中和紀念醫院
Kaohsiung Medical University Chung-Ho Memorial Hospital
人體試驗審查委員會
Institutional Review Board

807高雄市三民區自由一路100號
No. 100, Tzyou 1st Road, Kaohsiung 807, Taiwan
TEL： 886-7-3121101 ext.6646、886-7-3133525
FAX： 886-7-3221408

與正本相符

人體試驗期中報告同意證明書

計畫名稱：以專利大豆萃取液(ANT 1)改善雷射後患部泛紅、灼熱及最終整體美白效果之評估
IRB 編號：KMUHIRB-2014-07-01(I)
計畫編號：ANT1-01
計畫主持人：李書欣
協同主持人：吳益嘉、王惠民

本計畫案2015年第8次第一人體試驗審查委員會會議通過；西元2015年8月7日審查通過，特此 證明。**試驗有效期限自西元2015年7月11日至西元2016年7月10日**。(依照GCP規定，每屆滿一年，第一人體試驗委員會須重新審查是否可繼續進行。請於有效期限到期一個月前繳交期中報告以利本會進行審查)

高雄醫學大學附設中和紀念醫院
第一人體試驗審查委員會
主任委員：顏學偉

(高雄醫學大學附設中和紀念醫院 人體試驗審查委員會)

西 元 二 〇 一 五 年 八 月 七 日

Approval of Clinical Trial

7/Aug/2015

Protocol Title: *The skin rash control and cosmetic effect of Hydrolyezd Soybean Extract (ANT1) for skin care after LASER treatment*
Board Meeting/Approval dated: *2015-8th-IRB(I) / 7/Aug/2015*
IRB Number: *KMUHIRB-2014-07-01(I)*
Protocol Number: *ANT1-01*
Principal Investigator: *Su-Shin Lee*
Co- Investigator: *Yi-Chia Wu, Hui-Min Wang*

Above study is approved by the ***Institutional Review Board-I*** *on* ***2015/7/11*** *and valid till* ***2016/7/10****. The constitution and operation of this review board are according to the guidelines of GCP. According to GCP,* ***IRB-I*** *will have to review each clinical research case annually and decide whether continue it or not. Therefore, please send us your Annual Report one month before the expiry date.*
Sincerely yours,

Hsueh-Wei Yen
Hsueh-Wei Yen, M.D.
Chairman
Institutional Review Board- I
Kaohsiung Medical University
Chung-Ho Memorial Hospital

(Kaohsiung Medical University Institutional Review Board Chung-Ho Memorial Hospital)

99231040804021

高雄醫學大學附設中和紀念醫院 函(稿)

地址:80756高雄市三民區自由一路100號
承辦人：人體試驗審查委員會－吳珮瑄
電話：(07)3121101分機6646轉37
傳真：(07)3221408

受文者：如正副本行文單位
發文日期：中華民國 104 8. 17 日 (請勿填寫)
發文字號：高醫附委字第 1040003737 號
速別：
密等及解密條件或保密期限：
附件：見主旨

主旨：檢送本院整形外科李書欣醫師主持之「以專利大豆萃取液(ANT 1)改善雷射後患部泛紅、灼熱及最終整體美白效果之評估」(IRB 編號：KMUHIRB-2014-07-01(I)，計畫編號：ANT1-01）臨床試驗期中報告申請通過證明，請 核備。

說明：
一、本案於高醫第一人體試驗審查委員會2015年第8次(2015/8/7)會議審核通過，同意本臨床試驗之執行有效期限至：2016/7/10。
二、本院計畫主持人請於證書有效期限到期前一個月繳交期中報告；臨床試驗計畫結束或中止後三個月內繳交結案或中止報告。如發生不良反應事件時須依相關法規填妥表格送相關單位(IRB)，以上相關資料請自本院人委會網站中下載。

正本：衛生福利部食品藥物管理署(期中報告同意證明書影本)、本院計畫主持人：李書欣醫師(修正計畫同意書正本)
副本：僑陞生化技術股份有限公司(修正計畫同意書影本)(74147台南市新市區南科二路13號4樓)、協同主持人：吳益嘉、王惠民

院長：

承辦	會辦單位	決行
承辦單位：人體試驗審查委員會<br>承辦人：吳珮瑄 2015.8.12<br>電話：6646<br>單位主管：人委會執行秘書 蘇富敏 2015.8.12	人委會主任委員 顏學偉 2015.8	行政室/醫務秘書：許惠珍<br>副院長：分層負責授權副院長莊萬龍決行 2015.8.17<br>院長：已用印

發文： 打字： 校對： 監印： 8.18 檔號：

檔 號：
保存年限：

財團法人私立高雄醫學大學附設中和紀念醫院 函

地址：80756高雄市三民區自由一路100號
承辦人：人體試驗審查委員會－吳珮瑄
電話：(07)3121101分機6646轉37
傳真：(07)3221408

受文者：如正副本行文單位
發文日期：中華民國104年8月17日
發文字號：高醫附委字第1040003737號
速別：普通件
密等及解密條件或保密期限：
附件：見主旨

主旨：檢送本院整形外科李書欣醫師主持之「以專利大豆萃取液(ANT 1)改善雷射後患部泛紅、灼熱及最終整體美白效果之評估」(IRB編號：KMUHIRB-2014-07-01(I)，計畫編號：ANT1-01）臨床試驗期中報告申請通過證明，請核備。

說明：
一、本案於高醫第一人體試驗審查委員會2015年第8次(2015/8/7)會議審核通過，同意本臨床試驗之執行有效期限至：2016/7/10。
二、本院計畫主持人請於證書有效期限到期前一個月繳交期中報告；臨床試驗計畫結束或中止後三個月內繳交結案或中止報告。如發生不良反應事件時須依相關法規填妥表格送相關單位(IRB)，以上相關資料請自本院人委會網站中下載。

正本：衛生福利部食品藥物管理署(期中報告同意證明書影本)、本院計畫主持人：李書欣醫師(期中報告同意證明書正本)
副本：僑陞生化技術股份有限公司(期中報告同意證明書影本)、協同主持人：吳益嘉、王惠民

院長 鍾飲文

第1頁 共1頁

Figure S3. IACUC certificate of animal examination (IACUC number: 105-141).

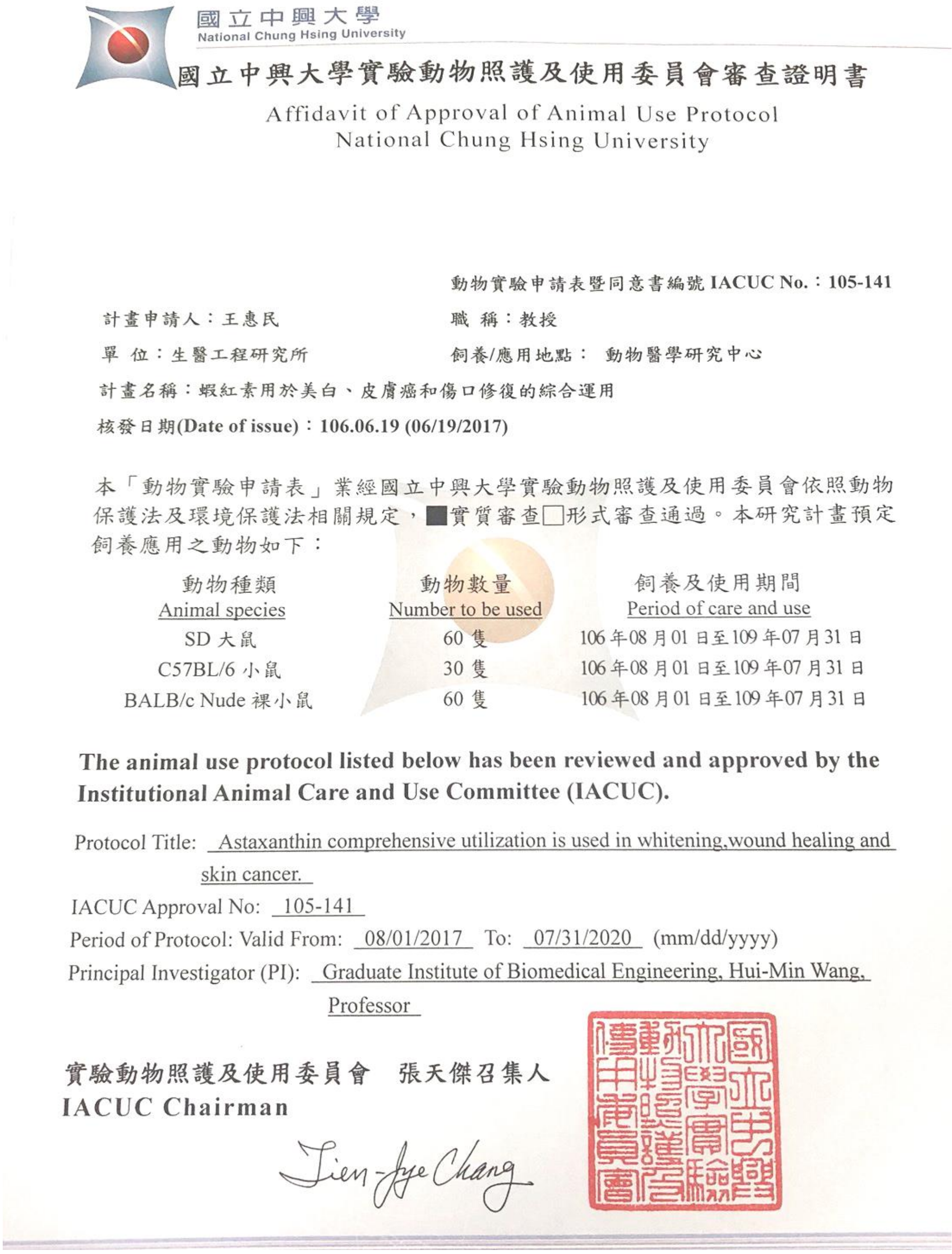

國立中興大學
National Chung Hsing University

國立中興大學實驗動物照護及使用委員會審查證明書

Affidavit of Approval of Animal Use Protocol
National Chung Hsing University

動物實驗申請表暨同意書編號 IACUC No.：105-141

計畫申請人：王惠民　　　　職　稱：教授

單　位：生醫工程研究所　　　　飼養/應用地點：　動物醫學研究中心

計畫名稱：蝦紅素用於美白、皮膚癌和傷口修復的綜合運用

核發日期(Date of issue)：106.06.19 (06/19/2017)

本「動物實驗申請表」業經國立中興大學實驗動物照護及使用委員會依照動物保護法及環境保護法相關規定，■實質審查□形式審查通過。本研究計畫預定飼養應用之動物如下：

動物種類 Animal species	動物數量 Number to be used	飼養及使用期間 Period of care and use
SD 大鼠	60 隻	106 年 08 月 01 日至 109 年 07 月 31 日
C57BL/6 小鼠	30 隻	106 年 08 月 01 日至 109 年 07 月 31 日
BALB/c Nude 裸小鼠	60 隻	106 年 08 月 01 日至 109 年 07 月 31 日

The animal use protocol listed below has been reviewed and approved by the Institutional Animal Care and Use Committee (IACUC).

Protocol Title: Astaxanthin comprehensive utilization is used in whitening,wound healing and skin cancer.

IACUC Approval No: 105-141

Period of Protocol: Valid From: 08/01/2017 To: 07/31/2020 (mm/dd/yyyy)

Principal Investigator (PI): Graduate Institute of Biomedical Engineering, Hui-Min Wang, Professor

實驗動物照護及使用委員會　張天傑召集人
IACUC Chairman

Tien-Jye Chang

Figure S4. TAP-nanoemulsion inhibited lung metastatic melanoma through induction of apoptosis related proteins by Western blotting verification.

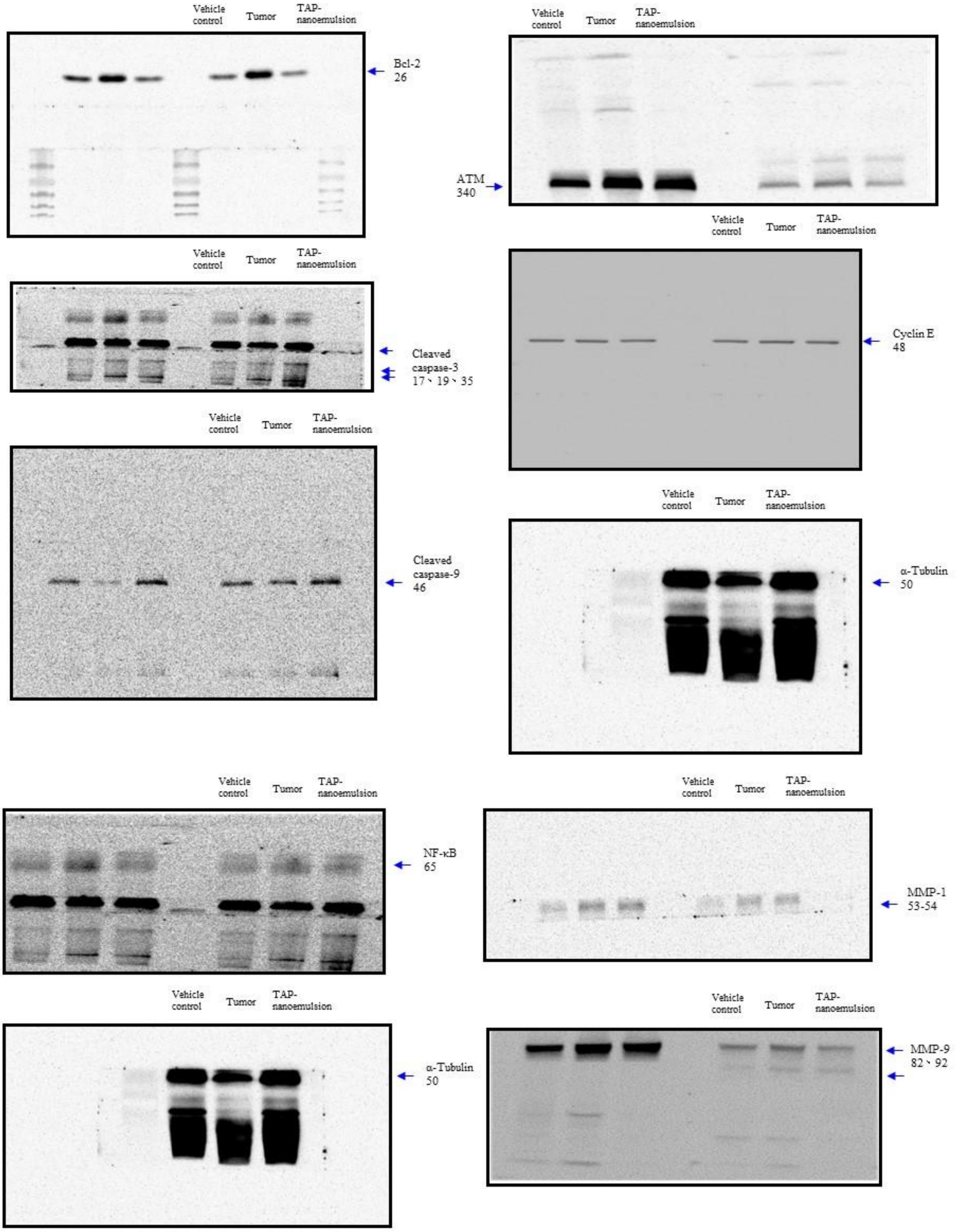

© 2020 by the authors. Submitted for possible open access publication under the terms and conditions of the Creative Commons Attribution (CC BY) license (http://creativecommons.org/licenses/by/4.0/).
